# Small-Scale Heterogeneity in Deep-Sea Nematode Communities around Biogenic Structures

**DOI:** 10.1371/journal.pone.0029152

**Published:** 2011-12-28

**Authors:** Christiane Hasemann, Thomas Soltwedel

**Affiliations:** Alfred Wegener Institute for Polar and Marine Research, Bremerhaven, Germany; King Abdullah University of Science and Technology, Saudi Arabia

## Abstract

The unexpected high species richness of deep-sea sediments gives rise to the questions, which processes produce and maintain diversity in the deep sea, and at what spatial scales do these processes operate? The idea of a small-scale habitat structure at the deep-sea floor provides the background for this study. At small scales biogenic structures create a heterogeneous environment that influences the structure of the surrounding communities and the dynamics of the meiobenthic populations. As an example for biogenic structures, small deep-sea sponges (*Tentorium semisuberites* Schmidt 1870) and their sedimentary environment were investigated for small-scale distribution patterns of benthic deep-sea nematodes. Sampling was carried out with the remotely operated vehicle Victor 6000 at the Arctic deep-sea observatory HAUSGARTEN. In order to investigate nematode community patterns sediment cores around three small sponges and corresponding control cores were analysed. A total of approx. 5800 nematodes were identified. The comparison of the nematode communities from sponge and control samples indicated an influence of the biogenic structure “sponge” on diversity patterns and habitat heterogeneity. The increased number of nematode species and functional groups found in the sediments around the sponges suggest that on a small scale the sponge acts as a gradient and creates a more divers habitat structure. The nematode community from the sponge sediments shows a greater taxonomic variance and species richness together with lower relative abundances of the species compared to those from control sediments. Obviously, the more homogeneous habitat conditions of the control sediments offer less micro-habitats than the sediments around the sponges. This seems to reduce the number of functional groups and species coexisting in the control sediments.

## Introduction

For many reasons, the deep sea is of great ecological interest. Living conditions are in an extreme range of many gradients for ecological stress (e.g. pressure or nutritional input) while at the same time the deep sea supports a rich and highly endemic fauna that varies in diversity on local, regional, and global scales [1]. Variability of biodiversity has been attributed among others to depth [2], latitude [3], [4] and to sediment structure [5], [6]. This high diversity of most deep-sea communities and how it is maintained are key questions in deep-sea ecology [e.g. 7]–[12]. Different, equilibrium (Stability Time Hypothesis) [7] and non-equilibrium hypotheses (Intermediate Disturbance Hypothesis [13], Spatial Time Hypothesis [14], Dynamic Equilibrium Hypothesis [15]) have been developed to explain the remarkably rich fauna of deep-sea soft-bottom habitats (cf. e.g. [16]).

This study captures some aspects of the non-equilibrium hypotheses that diversity is maintained by habitat heterogeneity created by biogenic structures and disturbances, respectively [17]. Benthic macro- and megafaunal organisms are able to create biogenic structures by altering the seafloor, constructing burrows and sediment mounds, leaving feeding traces and faecal pellets or building structures such as tubes. These sediment structures vary from millimetres to several centimetres in length; they disturb the sediment surface and lead to an increased nutrient flux across the sediment-water interface. Due to low hydrodynamic forces in the deep sea, these structures persist long enough to contribute to niche diversification for smaller benthic organisms. For shallow-water habitats such effects have frequently been demonstrated [18], but only a few studies investigated the impact of biogenic structures on small sediment-inhabiting organisms (bacteria to meiofauna) in the deep sea [19]–[25]. Another source for small-scale spatial heterogeneity are epibenthic sessile organisms like anemones, tunicates, hydroids and sponges. They alter near bottom flows and locally enhance particle deposition and erosion [20]. Compared to other benthic structures deep-sea sponges generate a specific physico-chemical microenvironment by modifying concentration gradients of solutes and particles across the sediment-water interface [26], [27]. As projecting structures they also passively interact with the near-bottom currents, thereby altering particle deposition and erosion rates [28]–[31]. Sponges provide a broad range of small-scale microhabitats and create a gradient of food supply for meiofauna organisms [32]. Results from these studies suggest that a specific meiofauna assemblage exists around small benthic structures.

In the present study we sampled nematode communities from sediments around centimetre-small sponges and from adjacent virtually homogeneous control sediments in the eastern Fram Strait at the deep-sea observatory HAUSGARTEN [33]. All marine free-living nematodes are members of the meiobenthos and usually comprise up to 95% of the metazoan meiofauna inhabiting deep-sea sediments [34]. An important attribute of their populations is the high species diversity by which they are represented in a wide variety of environments [35], usually in a magnitude higher than any other major taxon [36]. Deep-sea sediments contain reams of nematode species, and it seems that even small volumes of deep-sea sediments constitute a complex and diverse habitat for nematodes [37]. In the past, ecological research often addressed nematodes as a taxonomic unit of the meiofauna and considered them as a functionally homogeneous group. In fact, the various nematodes species are characterised by different morphological and functional traits. They are ecologically extremely heterogeneous and occupy different trophic positions in the benthic food web [35]. These factors make nematodes a useful tool for investigating both structural and functional diversity especially in the deep sea.

Studies on community structure are largely based on descriptions of assemblages using traditional diversity measures. The traditional way of analysing nematode community structure and diversity patterns is based on species abundance data, not take account of the phylogenetic relationships and the various ecological and biological characteristics of the nematode species. A description of biodiversity would be more ecological relevant if it is related to changes in functional diversity [38] and linked to the question what species do in an ecosystem [39]. Coupling of abundance-based, taxonomic and functional diversity can be a powerful tool in ecological research, although the relationship between them is still in an explorative field [40], [41]. In the present study abundance- and taxonomy-based diversity is analysed in addition to functional diversity. Therefore, diversity of the nematode community from the HAUSGARTEN observatory is not only examined as number of species, their relative density, and taxonomic relatedness of the nematode species [42], but is also described by means of functional traits of the species. Free-living nematodes show several morphological features that are thought to be ecological relevant [43]. To describe the functional structure of the nematode community each species was classified by three different morphological features. Buccal morphology [44] is one morphological trait that has been widely used in ecological work to classify nematodes by feeding types [45], [46]. Another functional classification is based on nematode tail shapes [47] as they are supposed to be important in locomotion and reproduction [43], [48]. Moreover nematodes show significant differences in body size and shape [49], [50]. Nematode length/width ratios describe another morphological adaption to a certain lifestyle [51]–[53]. In the present study each nematode species was classified into functional groups based on a combination of these morphological traits.

However, the functional group approach carries the risk of measuring the number of functional groups instead of functional diversity. Whilst the upper limit for the number of species is only restricted by the species pool, the upper limit of the number of functional groups used should be large enough to cover the entire functional trait spectrum represented in a community [54]. The functional groups used to describe functional diversity of the nematode communities at HAUSGARTEN are based on the connection of multiple morphological traits to minimise the loss of information, thus avoiding some of the problems associated with the functional group approach [55].

We examined the nematode community structure along a habitat gradient using traditional diversity measures, taxonomic properties and the functional group concept based on biological traits. These different components of biodiversity are described and compared to answer the following questions:

1. Does habitat heterogeneity affect nematode community composition on a local scale?
2. How differ the biodiversity patterns of the nematode communities from control samples compared to those from sponge samples?
3. Do the three different approaches (abundance-based, taxonomic, functional measures) provide different interpretable information on the nematode's biodiversity patterns?

## Materials and Methods

### Sampling

**Figure 1:**
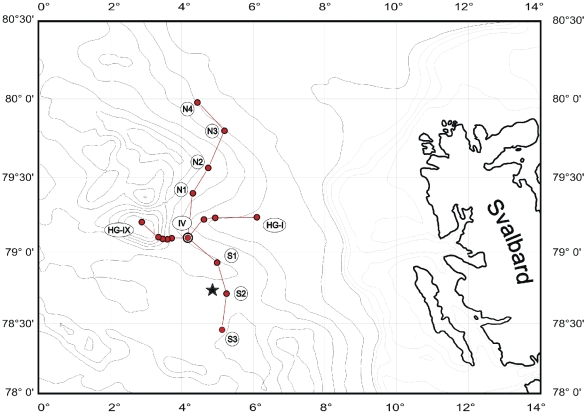
Location of HAUSGARTEN observatory. Bathymetric map of the HAUSGARTEN area with 16 permanent sampling sites (red dots). The star marks the sampling position of this study at 2300 m water depth.

Sampling was carried out in the eastern Fram Strait (Arctic Ocean) during an expedition with RV L'Atalante of the Institut Français de Recherche pour l'Exploration de la Mer (IFREMER) in summer 2001. In this area, at 79°N, 04°E, the German Alfred Wegener Institute for Polar and Marine Research (AWI) established the deep-sea observatory HAUSGARTEN [33]. The samples derived from the southern HAUSGARTEN area (78°45′N, 04°52′E) at 2300 m water depth (Figure 1). All necessary permits were obtained for the described field studies.

**Figure 2:**
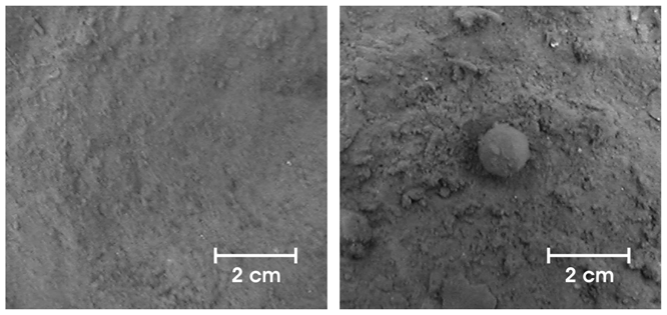
Photograph of the sediment surface of two push-cores. Control core (left picture) and sponge core with *Tentorium semisuberites* (right picture).

**Figure 3:**
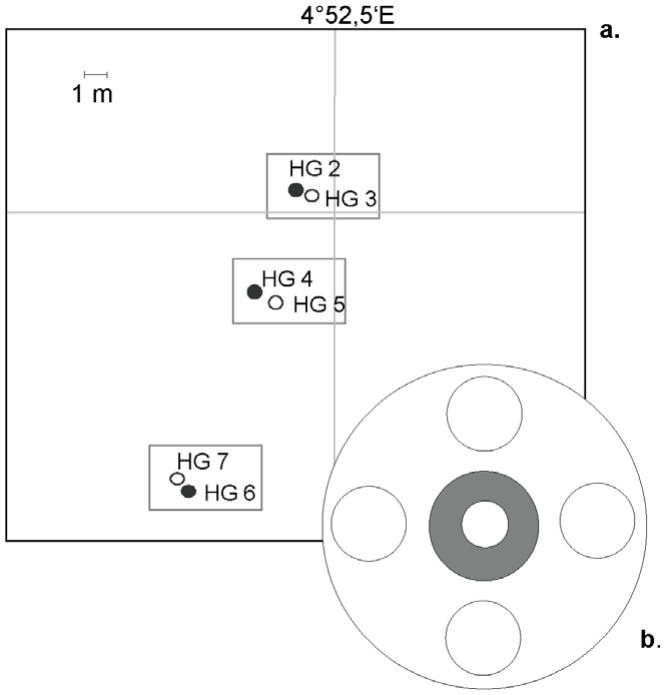
Close-up of the sampling site and sub-sampling of the sediment cores. (a.) Positions of the control cores (HG3, HG5, and HG7, open circles), and sponge cores (HG2, HG4, and HG6, closed circles). (b) Sub-sampling scheme of the sponge cores. Sub-sampling of the control cores corresponds to the sub-sampling scheme of the sponge cores.

Sediments surrounding small benthic sponges (1–2 cm in diameter) of the species *Tentorium semisuberites* (Schmidt 1870) were sampled by the French Remotely Operated Vehicle (ROV) Victor 6000, using Perspex™ push-cores (60 mm inner diameter) operated by the ROVs manipulator arm. Sediments with a virtually undisturbed surface and no protruding objects adjacent to the sponges were sampled to serve as controls (Figure 2). At each sampling point a pair of sediment cores (control and treatment, i.e. sediments with sponge) was taken. In total three pairs of sediment cores were sampled, at a distance of a few meters to each other (Figure 3a). The statistical model that guided our study was a randomised blocks design, with sampling sites as blocks (randomly chosen) and sponge and control cores as treatment.

To investigate small-scale distribution patterns of the nematodes, the uppermost 5 cm sediment-layers of the corer tubes were sub-sampled by means of small syringes (1.2 cm in diameter). From each corer tube sub-samples were taken at four positions around the sponge and below the sponge after removal. The sub-sampling scheme of the controls was similar to the sub-sampling of treatments (Figure 3b). Sub-samples were sliced into 1 cm layers and preserved in a 4% Formalin seawater solution. At the laboratory, the sediment layers were fractionated into five size-classes (500, 250, 125, 63, and 32 µm), which facilitates the separation of the nematodes from the sediment, and stained with Rose Bengal. For the determination of the nematodes to species level (or putative morphospecies), all individuals were prepared as permanent slides ([56], [57] and citations therein). Identification and measurements (width and length) of each individual has been done using light microscopy (up to 100-times magnification, with Nomarski optic). By means of a video camera (3-CCD Hitachi HV-C20) mounted to the microscope, a digital image database with about 10.000 detailed photos from about 600 individuals has been set up. For the investigation of the nematode communities all nematodes have been sorted from the sediments of 15 control sub-samples and 12 sponge sub-samples.

In order to allow sub-sampling at various positions around the sponges it was necessary to localise the sponges exactly in the centre of the push-cores. A precise positioning of the push-cores with the manipulator arm of the ROV was not always possible; therefore three sub-samples from the sponge cores are missing (one of each sponge core).

### Diversity measures

To measure and compare biodiversity patterns of the nematodes, taxonomic composition and functional traits of the nematode species, indicated by functional groups are described as complements to diversity as number of species and their relative abundance. For the calculation of the different diversity measures of the nematode community only the uppermost sediment layers of the sub-samples from control and sponge cores were taken into account.

#### Abundance-based diversity

To estimate abundance-based diversity of the nematode community, univariate indices were computed such as number of species (S), diversity (Shannon-Wiener index H' [58]), species richness (ES_(n)_ [59]) and evenness (Pielou's index J' [60]).

To test for differences in univariate diversity measures (J', ES_(n)_, H') between sampling sites (core pairs) and between treatments (control and sponge cores), we specified a nested design for the analysis of variance (nested ANOVA) with “Site” as random factor and “Site (Treatment)” as error term for the random factor “Site”. To achieve a balanced sample size 12 sub-samples from the sponge cores and the corresponding 12 sub-samples from the control cores were included in the analysis. Prior to analyses, homogeneity of variances was assessed with Cochran's C test. Tukey HSD multiple comparison test was used for pairwise comparisons. All indices were calculated after formulas as stated in [61]–[64].

#### Taxonomic diversity

[65] developed a group of diversity indices, based on the relatedness of species. These measures were designed from species lists (e.g. from certain sampling sites) and they are based on the taxonomic distances through the taxonomic classification tree between every pair of individuals [66]. To describe taxonomic relationships between species within the nematode communities of the control and sponge cores the **Av**erage **T**axonomic **D**istinctness (AvTD, Δ^+^) as a measure for the mean degree of relationship between the species, and the **Var**iation in **T**axonomic **D**istinctness (VarTD, Λ^+^) as a measure for the unevenness of the species distribution across the Linnean classification tree were calculated [65], [66]. We used seven taxonomic levels (species, genus, family, suborder, order, subclass and class), according to the classification described by [67] with equal step-length between the levels. With these taxonomic distinctness measures, data can be used in presence/absence form, and with different levels of sampling effort.

With the construction of taxonomic distinctness indices from species lists it is possible to test these indices for departure from expectation, based on a master taxonomy which comprises the stock of species in the region where the species were found at one locality [66], [68]. This requires a master list or inventory of species within defined taxonomic boundaries [66], [68]. The species composition of a sampled locality (observed diversity) can be compared with this master list (expected diversity). Discrepancies of observed values from expected values can be interpreted as loss or enhancement of diversity [66], [69]. The indices Δ^+^ and Λ^+^ are explained in detail in [66], [68]–[70].

Calculations of AvTD and VarTD indices as well as the abundance-based diversity measures were done using the PRIMER DIVERSE routines [71].

#### Functional diversity

To estimate functional diversity of the nematode communities, organisms were divided into life-form types by means of the structure of buccal cavity, tail shape and body shape. These characteristics are linked to feeding strategy, locomotion mode, and mobility of the nematodes. These connectable criteria are considered as morphological adaptation to a given life-style.

The nematodes were classified into feeding types due to the structure of their buccal cavities according to [44]. Although the validity of this approach has been discussed in different studies [45], [72]–[76], it remains an important and common tool to interpret the ecology of free-living nematode communities [43], [77]–[82] and is therefore also used in the present study.

The classification in different feeding types according to [44] is based on four classes: Groups IA and IB describe selective and non-selective deposit feeders without teeth. Groups IIA and IIB comprise epigrowth feeders as well as predators and omnivores with teeth (Table 1). The groups of deposit feeders (IA and IB) mainly consume bacteria and small-sized (IA) or larger-sized organic particles (IB). Epigrowth feeders (IIA) use their teeth to tap objects or scrap off surfaces for food. Predators and omnivores (IIB) also tap plant objects, but the most important feeding mode is predation or scavenging. They use their buccal armature to feed on nematodes or other small invertebrates.

**Table 1:** Functional classification of the nematodes based on morphological traits concerning buccal cavity structure, tail and body shape.

**1. Feeding types**		Feeding modus	Life-style
		*buccal cavity structure*	
	**Group IA**	selective deposit feeders	microvorous
		*minute buccal cavity, without buccal armatu*re	
	**Group IB**	none-selective deposit feeders	**↓**
		*various, bigger buccal cavity, without teeth or other buccal armature*	
	**Group IIA**	epigrowth feeders	**↓**
		*minute to medium-sized buccal cavity, small teeth*	
	**Group IIB**	predators & omnivores	carnivorous/omnivorous
		*large buccal cavity, solid teeth*	
**2. Tail-shape groups**		Tail shape	
	**Group I**	rounded	mobile
	**Group II**	short conical	**↓**
	**Group III**	short cylindrical	**↓**
		*a. cylindrical*	
		*b. cylindrical, with swollen tip*	
	**Group IV**	long conical	**↓**
		*a. conical*	
		*b. conical, pointed*	
	**Group V**	long cylindrical	hemisessile
		*a. cylindrical*	
		*b. elongated*	
		*c. filiform*	
**3. Body-shape groups**		Body shape	
		*l/w ratio*	
	**Group I**	stout	burrowing
		*0–10*	
	**Group II**	plump	**↓**
		*11–20*	
	**Group III**	thin	**↓**
		*21–40*	
	**Group IV**	slender	**↓**
		*41–80*	
	**Group V**	filiform	interstitial
		*81–16*0	

1. Feeding types according to Wieser (1953) based on buccal cavity structure.

2. Tail-shape groups (I–V) based on variety of tail shapes.

3. Body-shape groups (I–V) based on body **l**ength and body **w**idth ratio (l/w-ratio).

Another functional classification of the nematode species is based on the great variety of tail shapes [47], [48], [83]. The shape of the tail has an influence on locomotion, foraging and reproduction and is used as another aspect of nematode morphology for classification into functional groups [47], [43], [84]. In the present study a modification of the tail-shape groups described by [47], [48], [83] is used to classify the nematodes. The tail-shape types have been related to five main classes with up to three sub-divisions: rounded, short conical, short cylindrical, long conical and long cylindrical (Table 1). Nematodes of the first three groups with short tails are thought to have a mobile lifestyle, whereas long-tailed nematodes of the last two groups are less mobile or even hemisessile (cf. [85]).

Following [50], [51], the ratio between body length and width is assumed to be a morphological adaptation to a given life-style (interstitial *vs.* burrowing). In nematode species descriptions, amongst others, an indication of the De Man ratio *a* is given, which is the ratio of the total body length to maximum body diameter [83]. This ratio provides a quantitative measure of the body shape [51], [53]. In this study, a modified De Man ratio *a* (body length excluding filiform tails) is used by dividing the body length without tail by the body width. A small length/width ratio (l/w ratio) describes a plump body shape and a large l/w ratio means a more slender body shape. In line with examples for De Man ratio *a* given by [83], a total of five morphotypes based on different body shapes were distinguished (Table 1). The first two groups consist of nematodes with short and stout bodies. Group three to five comprise nematodes with long and slender bodies. Two important life-styles are separated by this grouping: burrowing species with short and stout bodies and interstitial species with long and slender bodies. According to [51], the long/thin body shape enables the nematodes to move swiftly through the sediment but may make them more vulnerable to predation. In contrast, nematodes meeting the stout morphotype may reduce this predation pressure, due to their greater width.

Each individual nematode could be described by a combination of morphological traits related to a functional feeding (**F**), tail (**T**) and body-shape (**B**) group. The nematodes were classified by these trait-combinations to functional **F_T_B** groups and assigned to a particular type of life-form. As these functional life-form types share certain morphological traits, the functional types were consistent with the guild concept [86], [87]. This approach implies, that species within the same guild (respectively of the same life-form type), also occupy the same ecological niches [87]. Thus the number of life-form types state to the variety of the existing ecological niches [88]. With four different feeding types and five different tail and body shape types, respectively, a total of 100 combinations are possible, representing 100 different functional F_T_B groups to describe functional diversity of the nematode community.

There are no standardised measures for quantifying functional diversity [89], but a commonly used measure for functional diversity is the number of functional groups represented by the species of an assemblage ([90] and citations therein). The diversity measure **F**unctional **G**roup **R**ichness (FGR) as expected number of ecological life-form types per species number was also used in this study. Some species occur in more than one life-form type (mainly because of their varying body width/length ratio). Therefore the number of “functional” species per core could be higher than the number of “taxonomical” species per core.

Shannon-Wiener diversity (H') and evenness (J') were calculated as univariate measures of functional diversity of the nematode community. As additional information about the variability of the distribution of the life-form types, the **C**oefficient of **V**ariation (CV), expressed as ratio between standard deviation (SD) and mean (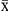) [91], was calculated for the functional structure of the nematode community.

The diversity measures for functional diversity were analysed using a randomised complete block analysis, with factors block “sampling site” (randomly chosen) and treatment “sponge/control core”. The response variable was diversity per core. The null hypothesis (H_0_) was that there are no differences in diversity between sponge and control samples per core, pooling across all possible blocks.

#### Life history strategy

According to [92]–[94], marine nematode taxa can be placed on an arbitrary c-p scale ranging from 1 for “colonisers” to 5 for “persisters”. Genera classified as colonisers have short live-cycles, high reproduction rates, high colonisation ability and are tolerant against various types of disturbance (r-strategist *sensu lato*). Genera classified as persisters have comparably long life-cycles, low colonisation ability, few offsprings and are more sensitive to disturbance (K-strategists *sensu lato*).

## Results

### Community structure

About 5800 nematodes were found in the sub-samples from control and sponge cores (0–5 cm sediment depth), corresponding to a mean density of 1467 ind./10 cm^2^. A total of 367 species were distributed over 92 genera and 31 families. 50% of all individuals were equally proportioned by three major groups. These were the family Desmoscolecidae with the genus *Desmoscolex*, Microlaimidae with the genus *Microlaimus*, and Monhysteridae with the genus *Thalassomonhystera* (see also Table S1).

Most of the nematodes were distributed in quite low densities over a multiplicity of species (high values of diversity H'(log_2_) and ES_(100)_, Table 2). The high evenness values for the species distribution indicated that none of the species showed a clear dominance within the nematode community. The high diversity rather reflected the large number of families and genera than a high diversity within the families and genera. Most of the nematode families consisted of few genera (≤8), 40% of the families were monogeneric. 92% of all genera were represented by less than 10 species; ca. 50% of the genera were monospecific. About a fifth of all families, genera and species were represented each by a single individual.

**Table 2:** Results of the diversity indices for the nematode communities in the sponge (HG2, HG4, HG6) and control cores (HG3, HG5, HG7) (0–1 cm sediment depth).

Diversity indicesSponge/Control core	*HG2/HG3*	*HG4/HG5*	*HG6/HG7*
**S**	126/105	158/138	104/129
***J'***	0.90/0.90	0.90/0.89	0.85/0.81
***ES_(100)_***	60/59	63/58	51/49
***H'*** **_(log2)_**	6.29/6.05	6.59/6.30	5.71/5.65

S: number of species, J': Pielou's index of evenness, ES_(100)_: expected number of species/100 individuals, H'_(log2)_: Shannon-Wiener diversity index.

### Abundance-based diversity

The diversity indices, calculated for the upper sediment layer of the control and sponge cores, showed higher values for the nematode communities from sponge cores than from control cores, except for species number of the core pair HG6/HG7. This core pair showed in fact the lowest values for the diversity indices (Table 1).

For the abundance-based diversity measures ES_(100)_ and H'(log_2_), results from the nested ANOVA with site as random factor showed significant differences (p = 0.022 and 0.039, respectively) between treatments nested within site from control and sponge cores. Species richness and Shannon-Wiener diversity of the sponge cores were significantly higher than in the control samples (Table 3). Evenness, however, showed no significant differences (p = 0.107) between control and sponge cores.

**Table 3:** Nested ANOVA analysis of differences in diversity indices (0–1 cm sediment depth) between sampling sites (Site) and control and sponge samples (Treatment).

	Source	SS	*df*	MS	*F*	*P*	Error term
**J'**	Site	0.005	2	0.003	2.837	0.203	Site (Treatment)
	Site (Treatment)	0.003	3	0.001	2.70	0.107	Residual
	Residual	0.012	12	0.001			
**ES_(100)_**	Site	787.836	2	393.918	1.996	0.281	Site (Treatment)
	Site (Treatment)	592.150	3	197.380	4.652	**0.022**	Residual
	Residual	509.130	12	42.430			
**H_(log2)_**	Site	1.471	2	0.735	2.397	0.239	Site (Treatment)
	Site (Treatment)	0.920	3	0.307	3.840	**0.039**	Residual
	Residual	0.959	12	0.080			

Bold values indicate significant differences at *p*<0.05.

### Taxonomic diversity

Table 4 outlines the results for the taxonomic diversity of the nematode communities from control and sponge cores. All samples tended to show reduced values for taxonomic distinctness. However, control cores (HG3, HG5, and HG7) predominantly showed lower values than expected from the master list for taxonomic variance, whereas the sponge cores (HG2, HG4, and HG6) rather showed increased variance values. All values for the taxonomic variance of the nematode community around the sponges were higher than expected. Increased variance values indicated a more uneven distribution of the species from the sponge cores across the taxonomic tree (cf. [66], [68]–[70]). A few genera became highly species-rich whilst a range of other genera were represented by only one or very few species.

**Table 4:** Taxonomic diversity indices and species number of the nematode communities from control and sponge cores (0–1 cm sediment depth).

Community	Number of species per core	Average taxonomic distinctness (Δ^+^)	Variation in taxonomic distinctness (Λ^+^)
Control cores (15 sub-samples)	219		
HG3 (5 sub-samples)	105	68.89 **(l)**	427.59 (l)
HG5 (5 sub-samples)	138	72.48 (exp)	473.72 (exp)
HG7 (5 sub-samples)	129	70.64 (l)	439.61 (l)
Sponge cores (12 sub-samples)	214		
HG2 (4 sub-samples)	126	71.54 (l)	461.28 (h)
HG4 (4 sub-samples)	159	70.83 **(l)**	480.23 (h)
HG6 (4 sub-samples)	106	71.95 (exp)	555.02 **(h)**

l = lower than expected; exp = as expected; h = higher than expected.

**In bold abbreviations**: index is significantly higher or lower than expected (95% significance level).

### Functional diversity

In total 52 out of 100 possible trait combinations or functional F_T_B groups, respectively, were found within the nematode community of this study. The nematode communities were dominated by four different ecological life-form types: small non-selective deposit feeders with a mobile, interstitial life-style; small non-selective deposit feeders with a less mobile, interstitial life-style; medium-sized selective deposit feeders with a mobile, burrowing life-style; large epigrowth feeders with a less mobile, burrowing life-style. The first trait combination dominated the sponge core HG2 (11%) and the second combination the corresponding control core HG3 (15%). These two trait combinations were the most genera-rich. 30 genera mainly belonging to the xyalids shared the first trait combination while a total of 16 mainly diplopeltid genera shared the second combination. The third trait combination dominated the core pair HG4/HG5 having 15% share each. This trait combination was mainly represented by the desmoscolecid genera *Tricoma* and *Desmoscolex*. The last trait combination dominated the sponge core HG6 with 16% and the corresponding control core HG7 with 22%. This trait combination was only represented by the microlaimid genus *Microlaimus*.

Highest numbers of ecological life-form types were found within the nematode community of the core pair HG4/HG5. Functional diversity (FGR_(100)_) and Shannon-Wiener diversity H'(log_2_) in HG4/HG5 were also found to be higher than for the remaining core pairs (Table 5). Functional evenness J', however, showed highest values for the core pair HG2/HG3. In contrast, highest values for the CV were found for the nematode community of core pair HG6/HG7.

**Table 5:** Univariate diversity measures and coefficient of variation for the ecological life-form types of control and sponge cores (0–1 cm sediment depth).

Sediment cores	S	N	J'	FGR_(100)_	H'_(log2)_	CV
HG2	31	162	0.89	28	4.43	0.23
HG3	26	117	0.88	25	4.11	0.14
HG4	36	199	0.85	29	4.39	0.36.
HG5	31	142	0.85	28	4.21	0.23
HG6	31	129	0.86	29	4.28	0.62
HG7	28	131	0.89	26	4.28	0.27

S = number life-form types per core, N = number of *functional* species per core, J': Pielou equitability index, FGR_(100)_: expected number life-form types per 100 *functional* species, H'_(log2)_: Shannon-Wiener diversity index, CV = coefficient of variation.

Comparing functional diversity (FGR_(100)_) and Shannon-Wiener diversity H'(log_2_) between sponge and control core of each core pair, values for the sponge core were equal to or higher than for the corresponding control core. Functional evenness (J') showed no clear trend. Control and sponge core of the pair HG4/HG5 showed the same values for evenness, whereas evenness of the remaining two core pairs was in one instance higher for the sponge core (HG2) and in one instance higher for the control core (HG7). CV-values of the sponge cores were always higher than for the controls.

At a 10% significance level, functional diversity FGR_(100)_ (*df* = 1; F = 4.566, p = 0.0501) and Shannon-Wiener diversity H'(log_2_) (*df* = 1; F = 3.373, p = 0.0637) of the sponge cores were higher than for control cores. All other functional diversity measures as well as the coefficient of variation showed no significant differences between control and sponge cores (neither at a 5% nor at a 10% significance level). There were no effects of blocks (sampling sites) although given the random blocks, except for the evenness (J'; *df* = 2; F = 4.92, p = 0.0240).

The sponge-associated sediments were dominated by colonisers (c-p value of 2), whereas more K-selected genera (c-p values of 3 and 4) prevailed the control sediments (Table 6). The percentage of nematode species whose individuals occur in more than one functional group was higher in the sponge-associated sediments (53%) than in the control sediments (37%, Table 7).

**Table 6:** Percentage of nematodes in life history categories (c-p values) in control and sponge samples (0–1 cm sediment depth).

c-p Value	Control	Sponge
c-p 2	39.8%	51.5%
c-p 3	20.6%	21.4%
c-p 4	39.4%	28.2%

c-p 2 = general opportunists, c-p 3 = more *K*-selected genera, c-p 4 = persisters.

**Table 7:** Percentage of species of control and sponge samples whose individuals are distributed over a different number of functional groups (0–1 cm sediment depth).

Number of functional groups per species	% Species	
	Control	Sponge
1	63	47
2	33	37
3	4	12
4	-	4

### Relationship between species diversity and functional diversity

**Figure 4:**
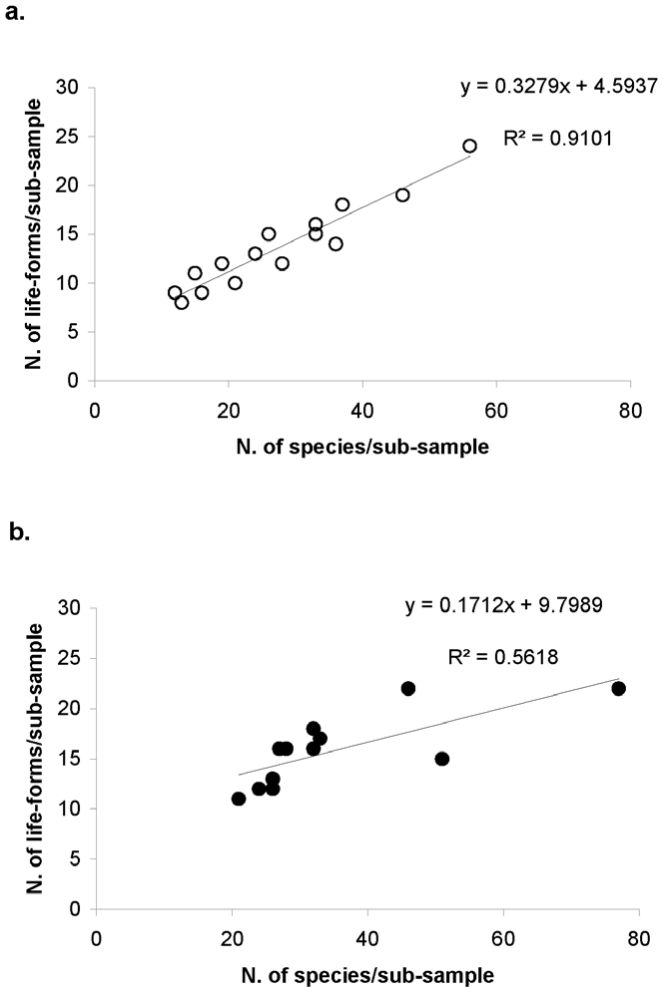
Relationship between functional diversity and species richness within the nematodes communities. (a) control and (b) sponge samples (sediment-depth 0–1 cm). R^2^ for a linear relationship. Results of regression: a. no. of cases: 15; p<0.001; df = 1.13; b. no. of cases: 12; p = 0.005; df = 1.10.

Abundance-based species richness was related to functional diversity as life-form richness. The number of ecological life-forms increased with increasing species number for control and sponge sub-samples (Figure 4). The species richness and life-form richness relationship revealed a linear increase for the nematode community from control samples (R^2^ = 0.9101). In the sponge cores species richness and life-form richness were related to a lower degree, expressed by a lower coefficient of determination (R^2^ = 0.5618). Beyond a certain level, increasing species numbers had no effect on the increase of life-form types. Altogether, life-form types of the nematode community from sponge samples comprised a higher number of species than from the controls.

## Discussion

### Data Quality

Ideally, sample size and number of sampling units should be optimised not only in relation to body size and quantitative abundance of the fauna but in particular to the statistical sampling design. However, in the deep sea benthic sampling is more costly, and time consuming, and technically more difficult than to obtain samples from shallower waters. Replicate or multipoint sampling over a small area, although statistically desirable, is therefore only rarely accomplished. Despite the above mentioned difficulties in sampling the deep sea and as a result of the targeted sampling that could only be achieved by using a ROV, we were able to obtain a sufficient set of samples which allows an accurate resolution of the nematode community patterns.

### Abundance-based Diversity

Habitat heterogeneity has been shown to modify benthic community characteristics like abundance, richness and diversity. According to [95] the majority of empirical studies find a positive relationship between habitat complexity and species diversity [96]–[100] (but see [101]). Heterogeneous environments are predicted to support more complex and diverse biological assemblages [102], [17]. In a competitive environment, spatial heterogeneity provides an additional axis, along which species can differ. This increases the possibility of species coexistence. The permanent heterogeneous environment around the sponges (see Figure 2) should rather promote the coexistence of many species than the dominance of particular species (cf. [103]). Whereas in a heterogeneous environment a community is composed by many different species in low individual densities, in homogeneous environments the community is dominated by less species in high density of the individuals (cf. [104], [105]). Against this backdrop it is expected that the community structure of the nematode assemblages is characterised by different species dominance patterns in the control samples and sponge samples, where higher diversities (species number and richness, evenness and Shannon-Wiener diversity) in the heterogeneous sediments around the sponges have been found. Control and sponge cores differ significantly in the diversity measures ES_(100)_ and H'(log_2_); these parameters are, however, interdependent. Regarding the uncorrelated parameters ES_(100)_ and J', no significant differences between the data collectives could be seen for the evenness, although evenness was higher in sponge than in control samples.

The diversity patterns of the nematode communities suggest that the abundance-occupancy relationship (relationship between density of individuals and distribution of nematode species) is related to the different environmental conditions and to the effects of different interspecific interactions in control sediments and around the sponges.

The partly significant different community structure in control and sponge samples in terms of diversity patterns described by the abundance-based measures merely displays a trend and can not be explained by a single mechanism (such as the Carrying Capacity Hypothesis [106], or the Aggregation Model of Coexistence [107]). Presumably, the underlying processes are insufficiently described by the traditional diversity indices alone, as they do not take into account the distances or differences between species or their trait dissimilarities. Taxonomic and/or functional diversity measures are more reliable community descriptors here as they drive more resp. additional information about the nematode community characteristics.

Although abundance-based diversity measures can easily be criticised on the grounds that they do not account for phylogenetic, taxonomic, and functional variability among species and are heavily dependent on sample size/effort, they are simple and intuitively sensible measures of species diversity.

### Taxonomic diversity

Describing the nematode community by taxonomic diversity leads to the following questions: “Do competing species need to be different in order to have a stable coexistence?” [108] and “Is there a limit to the similarity of coexisting species?” [109].

The coexistence of similar species often is interpreted by similar distribution patterns due to shared habitat requirements (similar fundamental niches, cf. [88], [110]–[112]). In the real world, however, no square centimetre of ground is exactly the same as the next and environmental conditions are subject to temporal variability [109]. Any force that interrupts the process of competitive exclusion may prevent extinction and enhance diversity [109].

Our comparison of sponge associated nematode assemblages with those from adjacent control sediments revealed that sub-samples from controls as well as from sponge cores tended to show low to expected values for taxonomic distinctness (AvTD). However, the control samples predominantly showed low values for taxonomic variance (VarTD), whereas sub-samples from sponge cores showed higher than expected variance values.

Obviously, coexistence of taxonomically similar species is promoted in the control cores, whereas more dissimilar species coexist in the sponge cores. More heterogeneous habitat conditions of the sediments around the sponges might operate as a force that interrupts the process of competitive exclusion and thus causes higher taxonomic variability and species richness of the sponge cores. Restricted available resources would have a stronger limiting effect on the control than on the sponge-associated communities and would cause a nematode community with reduced species richness in connection with a lower taxonomic variability.

Coexistence of the species within the sponge cores mainly depends on the response of the species to the heterogeneous environment. Thus the interactions between the species might be conditioned by the extent of ecological requirements overlap (or not overlap) along the relevant environmental parameters. This could lead to a negative correlation of taxonomic similarity and local coexistence within the sponge cores. The biogenic structure “sponge” creates a heterogeneous environment on a local scale compared to the regional level. On local scale, (taxonomically) similar competitors (low or rather expected AvTD-values) are being affected differently by the heterogeneous environment within the sponge cores, which is expressed by a more variable distribution of the nematode species across the genera (increased VarTD-values).

Compared to the regional level, species within the nematode community of the control cores tended to show a more even or rather expected distribution across the genera. This lower taxonomic variability could be determined by similar requirements concerning the same potentially limited resources. Local coexistence of species might be favoured, if species differ sufficiently in their relative effects (impact vector) as long as they do not differ too much in their relative response to the environmental conditions [113].

#### Are low AvTD values together with enhanced VarTD values characteristic for deep-sea nematode communities?

Provided that the taxonomic structure of the nematode community from the HAUSGARTEN area represents a composition, where species are distributed over various families (here: 31) but few orders (here: four), increased taxonomic variance indicates an uneven species distribution over genera. As all species of the nematode community belong to the same class, the greatest taxonomic differences between the species of the control and sponge cores occur on a low taxonomic level (family resp. genera level). Results from different studies show that deep-sea nematodes are usually distributed over rather a few characteristic genera, whereas the species distribution hardly overlaps [46], [114]–[118]. Taxonomic differences within deep-sea communities would predominantly appear on a low taxonomic level along with increased VarTD-values. Low AvTD values would be characteristic for these communities and may be a general feature of nematode communities from the deep sea.

An expected level of average taxonomic differences along with increased taxonomic variance of these nematode communities will, in contrast to what has been found by [68], not necessarily be a consequence of reduced habitat heterogeneity. Varying VarTD-values of the HAUSGARTEN nematode community more likely reflect the heterogeneity resp. homogeneity of environmental conditions, and therefore the extent of small-scale habitat heterogeneity. Results provided by [119] indicate that environmental variability has a stronger effect on the variation in taxonomic distinctness of a community than on the average taxonomic distinctness. The communities investigated by [119] show high VarTD values with variable environmental conditions and low VarTD values combined with reduced environmental variability (but see also [120]). The increased VarTD values of the nematode community from the sponge cores therefore could be interpreted as response to the more diverse habitat conditions of the sediments in the surroundings of the sponges, whereas the decreased taxonomic variance of the controls nematode community reflects more homogeneous habitat conditions of the control sediments.

In contrast to the abundance-based diversity measures, which treat all species as equivalent in value in their contribution to diversity, taxonomic distinctness measures quantify diversity as taxonomic relatedness of the species within a community, and thereby describe another dimension of diversity [121]. The fact that taxonomic distinctness measures are statistically independent of sampling effort [122] makes them attractive tools for investigating the structural complexity of an ecological community [123]. Finally, the question about (taxonomic) differences between species is not the only approach to understanding the coexistence of species under different environmental conditions. Species that are closely related evolutionarily are not necessarily morphologically or functionally similar [124]. After all, the key to understand the community structure of the nematodes is the knowledge how species coexist or to be more specific, which kind of niche differences exists and to what extent do niche differences occur within the control and sponge cores.

The taxonomic diversity measures demonstrated differences between the species of the nematode communities around the sponges and in the control sediments and they show that these differences are variable, but they do not define the character of theses differences (cf. [125], [126]).

### Functional diversity

Ecologists have used a variety of ways to define functional groups but there is no universal functional type classification for species in an ecosystem or a community. This applies especially to the classification of marine benthic invertebrates [127]. Functional classification depends on the objective of the investigation, the spatial scale (local to global), and the observed ecosystem processes or environmental factors [127], [128]. Therefore functional groups are arbitrary classifications within a comparative continuous characteristic space, like most categories used to simplify the real world.

The functional groups used to describe the HAUSGARTEN nematode community were consistent with the guild concept [86], [87]. Species within the same guild respectively of the same life-form type, also occupy the same ecological niches [87]. Thus the number of life-form types is an indication for the variety of the niches offered by an ecosystem [88].

Functional classification often has two distinct goals: (1) to investigate the response of species to environmental changes such as food or resource availability (functional response groups), and (2) to study the effect of species on properties of an ecosystem or community structures such as stability, resource dynamics or productivity (functional effect groups; [129], [130]). The nematode species of the southern HAUSGARTEN area have been classified into feeding types, tail-shape and body-shape groups as functional response groups. The combined functional approach to classify the nematode species into life-form types by means of F_T_B groups in contrast reflects functional effect groups. As a result hierarchical classification of the feeding types, tail-shape and body-shape groups has been performed [131].

The determined life-form types in connection with species richness of the nematode community were examined under aspects of functional redundancy (Redundant Species Hypothesis). The Insurance Hypothesis is closely associated with the concept of functional redundancy [132]. The higher the variance of the functional effect groups of a community, the less species are necessary (as lower species richness) to buffer an ecosystem [128]. Functional richness (as interspecific variance of responses to the environment) should contribute to the insurance effect, since higher functional richness increases the chance that at least some species respond differently to variable environmental conditions or disturbances [128]. Although species of a functional effect group should show by definition at least some degree of redundancy, they might respond differently to changes in the environment [128]. Thus, different functional response types nested within a functional effect group might be important in sustaining (long-term) functioning of an ecosystem [133]–[135]. Nonetheless, questions about the extent to which results from these terrestrial concepts can be extrapolated to the deep water still remain (cf. discussion in [136]–[139]).

#### Functioning of the nematode community from control and sponge cores

Diversity values for the functional structure of the nematode community only show differences at a 10% level between the communities of the control and sponge cores. Together with the higher CV values for the sponge communities, this might nevertheless be an indication not only for a higher functional diversity, but also for a higher functional divergence within the sponge-associated nematode community. Higher functional divergence induces a higher degree of niche partitioning and therewith a lower degree of resource competition. There is a high probability that a more efficient resource utilisation could cause a higher ecological community function within the sponge cores.

The relationship between species richness and functional diversity differs in the nematode communities from control and sponge samples. This relationship is linear within the control sediments: the higher the species richness, the greater the functional diversity (as number of functional groups). In contrast, the correlation between number of species and number of functional groups appears to be weaker with a lower slope within the community from sponge samples. This could be an indication that under homogeneous environmental conditions functional niches of the species do not overlap or rather less strong (control cores) than under heterogeneous environmental conditions, where niches of the species seem to overlap more broadly (sponge cores). These differences imply that for the community function of the sponge cores the species identity effect is less important than the functional identity of the species compared to control cores. The functional habitat structure of the sponge-associated sediments seems to favour a community in which diversity was not so much determined by the number of species but by the coexistence of individual species as well as their interactions. Increasing species richness was becoming possible due to a stronger differentiation of the available niche space. By contrast in the control cores an increase of species richness depends on the variety of ecological niches and was, therefore, related to an increasing functional diversity.

The extent to which certain communities are built by either functionally redundant or different species depends not only on species richness, but also on the abundance of opportunistic and conservative species (generalist or specialist species) within the functional groups [140]. The nematode community of the control cores is predominantly composed by conservative species or specialists, whereas within the sponge cores the portion of opportunistic genera or generalists is higher. The species of the sponge cores are more able to expand beyond their realised niches (cf. [141]) and the capability of functional compensation (e.g. loss of species) is higher than that of the control cores (cf. [142]).

Compared to the sponge-associated nematode community, the community of the controls is defined by a lower number of functional groups and number of species within the functional groups along with a lower rate of species, whose certain individuals occur in more than one functional group. This is an indication that under the more homogeneous environment of the control sediments less (functional) niche space is available. The functional niches are narrower but more different, and there is less empty niche space. This could mean that less interaction takes place between the species of the control cores.

Changing environmental conditions (e.g. by disturbance or resources availability) would then probably be associated with changes in diversity and subsequently result in changes of the ecological community function (if changes of species richness also result in changes of functional diversity). Communities with weak species interactions might show a positive relationship between species diversity and extent of ecological function (cf. [140]). Species loss could not be compensated for by the remaining species and would represent the loss of a functional group, which subsequently could have a major impact on ecological processes within the community (cf. [143] and citations therein).

By contrast, overall more functional groups occur within the sponge cores. The particular functional groups contain more species and the portion of species, whose individuals occur in more than one functional group, is higher in the sponge cores compared to the control cores. This is a further indication that the heterogeneous environmental conditions in the sponge-associated sediments are quite likely to provide a larger functional niche space and niche overlapping is thus stronger than in the control cores.

Changing environmental conditions could probably be compensated by the nematode community from sponge cores without changes in diversity or variance in diversity would at most be damped (cf. [144] and citations therein). The stronger interaction between the species of the sponge-associated community could lead to intensified interspecific competition. Reduced abundance of particular species would lead to competitive release and could be compensated for by higher abundances of other species [144]–[147]. This negative feedback between species reduces not only the variance of total abundance within the community but also the variability in the ecological function of the community as a whole [140], [143]. Nevertheless, reduced biodiversity would have a negative effect on the Natural Insurance Capital [148] of the nematode community from sponge cores and thus, also a negative effect on the potential for functional compensation within the nematode community. There is a high probability that the function of the sponge-associated nematode community depends more strongly on biodiversity than the community of the control cores.

Results for functional diversity showed a higher level of ecological function within the nematode community from the sponge cores (higher functional diversity and more interaction between the species) compared to control cores. As long as the degree of environmental heterogeneity does not reach a point where the individuals of different species no longer interact, these mechanisms cause a certain redundancy within the functional groups.

Functional diversity is an important and rather complex component of biodiversity, yet in comparison to taxonomic diversity, methods of quantifying functional diversity are less well developed [89]. When used as a measure of functional diversity, species richness implicitly assumes that all species are equally different (addition of any species to a community will increase functional diversity by one unit) and that the contribution of each species to functional diversity is independent of species richness [149]. Functional group richness also has limitations. One significant disadvantage is perhaps the largely arbitrary decision about the extent at which differences are excluded [85], [150]. It assumes that species within groups are functionally identical [142] and that all pairs of species drawn from different functional groups are equally different. As a consequence we produced a classification based on different functional traits to minimise the loss of information.

A combination of multiple disequilibrium processes and mechanisms, interacting in complex ways, affects the nematode community structure in different ways in control and sponge-associated sediments. Under the rather homogeneous environmental conditions of the control sediments species diversity is associated with an increase of ecological different groups. However, the number of species and the functional richness is lower than under comparably heterogeneous environmental conditions of sponge-associated sediments.

In deep-sea environments spatial (and temporal) heterogeneity persists on a smaller spatial scale and over longer time periods than in comparable shallow water environments (e.g. [16], [151], [152]). Lower production rates and slower population growth rates than in comparable shallow water habitats lead to a certain competitive similarity of the species and contribute to extend time periods for competitive exclusion. Based on these assumptions there is a high probability that environmental heterogeneity on small spatial scales plays a more important role in maintaining diversity of the communities than productivity and competitive exclusion [136].

Comparing the different approaches to describe diversity, we demonstrated that at least for local scale studies the traditional species-level approach alone is less conclusive. Although our examination of the nematode communities from control and sponge-associated sediments is only a snapshot, our findings show that more emphasis should be placed on the various aspects of diversity (e.g. as physiological, morphological, behavior related and other traits which are represented by the species of a community), to evaluate the effects of diversity on community or ecosystem functioning and to identify the underlying mechanisms and processes on a spatial (and temporal) scale.

## Supporting Information

Table S1 Nematode genera and families identified in control and sponge samples (mean values including standard deviation, 0–5 cm sediment depth) and their functional classification.
(DOC)
