# Supplementary material for: Small-Scale Heterogeneity in Deep-Sea Nematode Communities around Biogenic Structures
Source: PLoS One. 2011 Dec 28;6(12):e29152. doi: 10.1371/journal.pone.0029152 (PMC3247241; doi:10.1371/journal.pone.0029152)
Supplement: Table S1 — Nematode genera and families identified in control and sponge samples (mean values including standard deviation, 0–5 cm sediment depth) and their functional classification. (DOC) [file pone.0029152.s001.doc]

Appendix SI. Genera list and functional classification of the nematodes from control and sponge samples.

|  | **F_T_B group** | **control (ind./10 cm²) (%/F_T_P group)** | **sponge (ind./10 cm²) (%/F_T_P group)** | **c-p value** |
| --- | --- | --- | --- | --- |
| **Enoplidae** |  |  |  |  |
| *Enoplus* Dujardin 1845 |  | 0.6(±2.3) |  | 5 |
|  | 2B_IV_III | 100 |  |  |
| **Thoracostomopsidae** |  |  |  |  |
| *Enoploides* Ssaweljev 1912 |  |  | 1.3(±3.0) | 2 |
|  | 2B_IV_II |  | 33.3 |  |
|  | 2B_IV_III |  | 66.7 |  |
| *Enoplolaimus* De Man 1893 |  | 0.6(±2.3) | 1.7(±3.4) | 2 |
|  | 2B_IV_III | 100 | 66.7 |  |
|  | 2B_IV_IV |  | 33.3 |  |
| *Epacanthion* Wieser 1953 |  | 0.2(±0.8) |  | 2 |
|  | 2B_II_II | 100 |  |  |
| *Paramesacanthion* Wieser 1953 |  | 0.6(±2.3) | 2.5(±3.9) | 2 |
|  | 2B_IV_III | 100 | 100 |  |
| **Phanodermatidae** |  |  |  |  |
| *Micoletzkyia* Ditlevsen 1926 |  | 0.2(±0.8) |  | 4 |
|  | 1A_IV_II | 100 |  |  |
| **Anticomidae** |  |  |  |  |
| *Anticoma* Bastian 1865 |  | 0.2(±0.8) | 0.3(±0.9) | 2 |
|  | 1A_II_IV | 100 |  |  |
|  | 1A_V_II |  | 100 |  |
| **Ironidae** |  |  |  |  |
| *Dolicholaimus* De Man 1888 |  | 0.6(±2.3) | 0.3(±0.9) | 2 |
|  | 2B_V_III | 100 | 100 |  |
| *Syringolaimus* De Man 1888 |  | 9.5(±8.7) | 9.3(±6.9) | 4 |
|  | 2B_IV_III | 12 | 5.3 |  |
|  | 2B_V_III | 88 | 63.2 |  |
|  | 2B_V_IV |  | 26.3 |  |
|  | 2B_V_V |  | 5.3 |  |
| **Leptosomatidae** |  |  |  |  |
| *Gen*. indet. |  | 2.2(±3.8) | 2.7(±5.6) | 5 |
|  | 1A_IV_III | 40 | 80 |  |
|  | 1A_IV_IV | 60 | 20 |  |
| **Oxystominidae** |  |  |  |  |
| *Halalaimus* De Man 1888 |  | 57.3(±33.5) | 68.5(±31.4) | 4 |
|  | 1A_IV_III | 2.3 | 2.5 |  |
|  | 1A_IV_IV | 3.1 | 3.3 |  |
|  | 1A_V_II |  | 1.6 |  |
|  | 1A_V_III | 39.8 | 35.2 |  |
|  | 1A_V_IV | 53.1 | 54.1 |  |
|  | 1A_V_V | 1.6 | 3.3 |  |
| *Litinium* Cobb 1920 |  | 5.1(±7.3) | 7.2(±11.7) | 4 |
|  | 1A_I_III | 40 | 72.7 |  |
|  | 1A_I_IV | 60 | 27.3 |  |
| *Oxystomina* Filipjev 1921 |  | 8.3(±7.8) | (8.3(±5.8) | 4 |
|  | 1A_IV_II |  | 5.9 |  |
|  | 1A_IV_III | 33.3 | 17.6 |  |
|  | 1A_IV_IV | 61.1 | 64.7 |  |
|  | 1A_IV_V | 5.6 | 5.9 |  |
|  | 1A_V_IV |  | 5.9 |  |
| *Thalassoalaimus* De Man 1893 |  | 2.0(±4.9) |  | 4 |
|  | 1A_II_III | 75 |  |  |
|  | 1A_II_IV | 25 |  |  |
| *Wieseria* Gerlach 1956 |  | 6.7(±8.4) | 7.7(±5.9) | 4 |
|  | 1A_IV_II |  | 7.7 |  |
|  | 1A_IV_III |  | 15.4 |  |
|  | 1A_IV_IV | 7.1 |  |  |
|  | 1A_V_III | 14.3 | 30.8 |  |
|  | 1A_V_IV | 71.4 | 30.8 |  |
|  | 1A_V_V | 7.1 | 15.4 |  |
| *Gen*. indet |  | 1.1(±3.3) | 1.1(±3.7) | 4 |
|  | 1A_V_IV | 80 | 100 |  |
|  | 1A_V_V | 20 |  |  |
| **Oncholaimidae** |  |  |  |  |
| *Meyersia* Hopper 1967 |  | 2.0(±3.6) | 1.5(±3.0) | 4 |
|  | 2B_IV_III | 100 | 50 |  |
|  | 2B_IV_III |  | 50 |  |
| **Enchelidiidae** |  |  |  |  |
| *Bathyeurystomina* Lambshead & Platt 1979 |  | 0.8(±2.4) |  | 4 |
|  | 2B_V_III | 50 |  |  |
|  | 2B_V_IV | 50 |  |  |
| *Eurystomina* Filipjev 1921 |  | 0.6(±2.3) |  |  |
|  | 2B_IV_II | 100 |  |  |
| **Trefusiidae** |  |  |  |  |
| *Trefusia* De Man 1893 |  |  | 0.7(±2.6) | 4 |
|  | 1A_V_IV |  | 100 |  |
| **Benthimermithidae*** |  |  |  |  |
| *Benthimermis* Petter 1980 |  | 0.6(±2.3) |  | parasite |
|  | red_I_III | 100 |  |  |
| **Chromadoridae** |  |  |  |  |
| *Acantholaimus* Allgén 1933 |  | 84.1(±35.8) | 74.3(±30.5) | 3 |
|  | 2A_IV_I | 1.1 | 0.7 |  |
|  | 2A_IV_II | 40.2 | 34.6 |  |
|  | 2A_IV_III | 7.8 | 12.5 |  |
|  | 2A_IV_IV | 0.6 |  |  |
|  | 2A_V_I | 1.1 |  |  |
|  | 2A_V_II | 33 | 33.1 |  |
|  | 2A_V_III | 15.6 | 16.2 |  |
|  | 2A_VIV | 0.6 | 2.9 |  |
| *Chromadora* Bastian 1865 |  | 27.2(±22.6) | 24.4(22.9) | 3 |
|  | 2A_II_II | 22.6 | 21.7 |  |
|  | 2A_II_III | 75.8 | 78.3 |  |
|  | 2A_II_IV | 1.6 |  |  |
| *Chromadorella* Filipjev 1918 |  |  | 1.0(±2.6) | 3 |
|  | 2A_II_II |  | 100 |  |
| *Trochamus* Boucher & Bovée 1972 |  | 14.7(±11.4) | 15.9(±10.8) | 3 |
|  | 2A_II_II | 12.5 | 21.4 |  |
|  | 2A_II_III | 9.4 | 14.3 |  |
|  | 2A_V_III | 34.4 | 35.7 |  |
|  | 2A_V_IV | 43.8 | 28.6 |  |
| **Comesomatidae** |  |  |  |  |
| *Cervonema* Wieser 1954 |  | 30.4(±17.9) | 22.9(±13.1) | 2 |
|  | 1A_II_II | 4.5 | 4.8 |  |
|  | 1A_II_III | 20. | 11.9 |  |
|  | 1A_IV_II | 15.4 | 9.5 |  |
|  | 1A_IV_III | 60 | 66.7 |  |
|  | 1A_IV_IV |  | 7.1 |  |
| *Comesomoides* Gourbault 1980 |  | 1.2(±2.8) | 4.2(±5.8) | 2 |
|  | 1B_IV_II | 50 | 14.3 |  |
|  | 1B_IV_III | 50 | 85.7 |  |
| *Laimella* Cobb 1920 |  | 0.6(±2.3) | 0.7(±2.6) | 2 |
|  | 2A_IV_III | 100 |  |  |
|  | 2A_IV_IV |  | 100 |  |
| *Pierrickia* Vitiello 1970 |  | 1.4(±3.1) |  | 2 |
|  | 1A_IV_II | 33.3 |  |  |
|  | 1AIV_II | 66.7 |  |  |
| *Sabatieria* Rouville 1903 |  | 3.2(±5.5) | 3.9(±4.4) | 3 |
|  | 1B_III_II | 16.4 | 1.9 |  |
|  | 1B_III_III | 69.1 | 83 |  |
|  | 1B_III_IV | 1.8 |  |  |
|  | 1B_IV_III | 12.7 | 15.1 |  |
| **Ethmolaimidae** |  |  |  |  |
| *Filitonchus* Platt 1982 |  | 0.6(±2.3) |  | 3 |
|  | 1A_IV_III | 100 |  |  |
| **Cyatholaimidae** |  |  |  |  |
| *Cyatholaimus* Bastian 1865 |  | 0.6(±2.3) | 0.7(±2.6) | 3 |
|  | 2A_IV_II | 100 |  |  |
| *Longicyatholaimus* Micoletzky 1924 |  | 7.3(±8.2) | 7.5(±8.4) | 3 |
|  | 2A_V_II |  | 7.7 |  |
|  | 2A_V_III | 53.3 | 53.8 |  |
|  | 2A_V_IV | 40 | 38.5 |  |
|  | 2A_V_V | 6.7 |  |  |
| *Minolaimus* Vitiello 1970 |  | 1.6(±3.1) | 4.5(±7.1) | 3 |
|  | 1B_IV_II |  | 12.5 |  |
|  | 1B_IV_III | 75 | 62.5 |  |
|  | 1B_IV_IV | 25 | 25 |  |
| *Paracyatholaimus* Micoletzky 1922 |  | 2.0(±3.3) | 1.5(±3.0) | 2 |
|  | 2A_V_III | 66.7 | 50 |  |
|  | 2A_V_IV | 33.3 | 50 |  |
| *Pomponema* Cobb 1970 |  | 7.4(±5.9) | 3.2(±5.7) | 4 |
|  | 2B_IV_II | 82.4 | 60 |  |
|  | 2B_IV_III | 17.6 | 40 |  |
| **Selachnematidae** |  |  |  |  |
| *Gammanema* Cobb 1894 |  |  | 0.7(±2.6) | 3 |
|  | 2B_V_IV |  | 100 |  |
| *Halichoanolaimus* De Man 1886 |  | 3.2(±5.4) | 0.7(±2.6) | 3 |
|  | 2B_IV_II | 50 | 100 |  |
|  | 2B_V_II | 33.3 |  |  |
|  | 2B_V_III | 16.7 |  |  |
| *Richtersia* Steiner 1916 |  | 0.6(±2.3) |  | 3 |
|  | 1B_II_I | 100 |  |  |
| **Desmodoridae** |  |  |  |  |
| *Molgolaimus* Ditlevsen 1921 |  | 3.5(±5.6) | 5.8(±8.8) | 3 |
|  | 1A_II_II | 66.7 |  |  |
|  | 1A_II_III | 33.3 | 100 |  |
| *Spirinia* Gerlach 1963 |  | 11.2(±22.5) | 16.7(±16.0) | 3 |
|  | 2A_II_II | 100 | 93.5 |  |
|  | 2A_II_III |  | 6.5 |  |
| **Microlaimidae** |  |  |  |  |
| *Microlaimus* De Man 1880 |  | 238.1(±346.7) | 261.4(±391.1) | 2 |
|  | 2A_II_I | 0.5 | 0.5 |  |
|  | 2A_II_II | 83.2 | 80.6 |  |
|  | 2A_II_III | 16.3 | 18.9 |  |
| **Leptolaimidae** |  |  |  |  |
| *Alaimella* Cobb 1920 |  | 5.7(±7.1) | 4.5(±4.7) | 3 |
|  | 1A_II_II | 36.4 | 20 |  |
|  | 1A_II_III | 63.6 | 80 |  |
| *Antomicron* Cobb 1920 |  |  | 1.0(±2.6) | 3 |
|  | 1A_IV_II |  | 50 |  |
|  | 1A_IV_III |  | 50 |  |
| *Camacolaimus* De Man 1889 |  | 5.1(±7.3) | 13.6(±22.1) | 3 |
|  | 2A_II_III | 40 | 57.1 |  |
|  | 2A_II_IV | 60 | 42.9 |  |
| *Diodontolaimus* Southern 1914 |  | 0.6(±2.5) | 0.3(±0.9) | 3 |
|  | 2A_II_III | 66.7 | 100 |  |
|  | 2A_II_IV | 33.3 |  |  |
| *Leptolaimoides* Vitiello 1971 |  | 3.0(±5.1) | 5.2(±7.8) | 3 |
|  | 1A_IV_III | 77.7 | 77.7 |  |
|  | 1A_IV_IV | 22.3 | 22.2 |  |
| *Leptolaimus* De Man 1876 |  | 30.8(±22.7) | 65.2(±37.0) | 2 |
|  | 1A_II_II | 23.7 | 24.3 |  |
|  | 1A_II_III | 72.9 | 72.9 |  |
|  | 1A_II_IV | 3.4 | 2.8 |  |
| *Procamacolaimus* Gerlach 1954 |  | 0.6(±2.3) | 0.3(±0.9) | 3 |
|  | 2A_II_III | 100 |  |  |
|  | 2A_II_IV |  | 100 |  |
| **Aegialoalaimidae** |  |  |  |  |
| *Aegialoalaimus* De Man 1907 |  | 18.3(±12.7) | 25.9(±13.3) | 4 |
|  | 1A_I_II | 7.7 | 6.4 |  |
|  | 1A_I_III | 92.3 | 93.6 |  |
| *Cyartonema* Cobb 1920 |  | 3.6(±5.6) | 1.3(±2.7) | 3 |
|  | 1A_I_II | 12.5 |  |  |
|  | 1A_I_III | 87.5 | 100 |  |
| **Diplopeltoididae** |  |  |  |  |
| *Diplopeltoides* Gerlach 1962 |  | 6.2(±5.5) | 9.4(±8.2) | 3 |
|  | 1A_I_I | 6.7 |  |  |
|  | 1A_I_II | 73.3 | 33.3 |  |
|  | 1A_I_III | 13.3 | 40 |  |
|  | 1A_II_II |  | 6.7 |  |
|  | 1A_II_III | 6.7 | 20 |  |
| *Gen*. indet |  | 2.0(±3.6) | 3.3(±6.0) | 3 |
|  | 1A_IV_III | 75 | 55.6 |  |
|  | 1A_IV_IV | 25 | 44.4 |  |
| **Tubolaimoididae** |  |  |  |  |
| *Chitwoodia* Gerlach 1956 |  | 1.2(±3.1) |  | 3 |
|  | 1A_IV_III | 100 |  |  |
| **Ceramonematidae** |  |  |  |  |
| *Pselionema* Cobb 1933 |  | 3.2(±5.5) | 3.9(±4.4) | 3 |
|  | 1A_II_III | 100 | 100 |  |
| **Desmoscolecidae** |  |  |  |  |
| *Desmolorenzia* Freudenhammer 1975 |  | 0.6(±2.3) | 0.8(±2.0) | 4 |
|  | 1A_III_I | 100 | 33.3 |  |
|  | 1A_III_II |  | 33.3 |  |
|  | 1A_III_III |  | 33.3 |  |
| *Desmoscolex* Claparéde 1863 |  | 46.8(±25.5) | 61.2(42.4) | 4 |
|  | 1A_III_I | 97.9 | 84.2 |  |
|  | 1A_III_II | 2.1 | 12.9 |  |
|  | 1A_III_III |  | 3 |  |
| *Greeffiella* Cobb 1922 |  |  | 0.3(±0.9) | 4 |
|  | 1A_III_I |  | 100 |  |
| *Quadricoma* Filipjev 1922 |  | 10.3(±11.7) | 6.6(±7.7) | 4 |
|  | 1A_III_I | 45 | 55.6 |  |
|  | 1A_III_II | 55 | 44.4 |  |
| *Tricoma* Cobb 1893 |  | 199.8(±125.7) | 189.9(±91.0) | 4 |
|  | 1A_III_I | 76 | 71.6 |  |
|  | 1A_III_II | 24 | 26.3 |  |
|  | 1A_III_III |  | 1.5 |  |
|  | 1A_III_IV |  | 0.6 |  |
| **Monysteridae** |  |  |  |  |
| *Thalassomonhystera* Jacobs 1987 |  | 266.1(±147.9) | 227.4(±100.0) | 2 |
|  | 1B_II_II | 5.9 | 6.3 |  |
|  | 1B_II_III | 74.1 | 58.4 |  |
|  | 1B_II_IV | 0.8 | 1 |  |
|  | 1B_IV_I |  | 0.3 |  |
|  | 1B_IV_II | 0.3 | 1.5 |  |
|  | 1B_IV_III | 7.6 | 11.7 |  |
|  | 1B_IV_IV | 1 | 1.8 |  |
|  | 1B_V_II | 0.3 | 0.8 |  |
|  | 1B_V_III | 6 | 14.5 |  |
|  | 1B_V_IV | 3.9 | 3.8 |  |
| **Xyalidae** |  |  |  |  |
| *Amphimonhystera* Allgén 1929 |  | 12.4(±15.8) | 18.9(±16.2) | 2 |
|  | 1B_IV_II | 7.4 | 2.9 |  |
|  | 1B_IV_III | 74.1 | 70.6 |  |
|  | 1B_IV_IV | 17.8 | 20.6 |  |
|  | 1B_V_II | 3.7 |  |  |
|  | 1B_V_III |  | 5.9 |  |
| *Amphimonhystrella* Timm 1961 |  | 0.6(±2.3) |  | 2 |
|  | 1B_III_III | 100 |  |  |
| *Daptonema* Cobb 1920 |  | 11.4(±10.4) | 18.1(±21.1) | 2 |
|  | 1B_IV_II | 17.2 | 17.2 |  |
|  | 1B_IV_III | 72.4 | 75.9 |  |
|  | 1B_IV_IV |  | 6.9 |  |
|  | 1B_V_III | 10.3 |  |  |
| *Gnomoxyala* Lorenzen 1977 |  | 12.1(±10.2) | 14.2(±12.1) | 2 |
|  | 1B_IV_II | 11.1 | 14.8 |  |
|  | 1B_IV_III | 85.2 | 77.8 |  |
|  | 1B_IV_IV | 3.7 | 7.4 |  |
| *Linhystera* Juario 1974 |  | 10.9(±14.3) | 8.2(±13.7) | 2 |
|  | 1A_IV_II | 14.3 |  |  |
|  | 1A_IV_III | 42.9 | 41.7 |  |
|  | 1A_IV_IV | 42.9 | 50 |  |
|  | 1A_IV_V |  | 8.3 |  |
| *Paramonhystera* Steiner 1916 |  | 2.0(±4.9) | 10.6(±15.8) | 2 |
|  | 1B_IV_III | 100 | 60 |  |
|  | 1B_IV_IV |  | 40 |  |
| *Rhynchonema* Cobb 1920 |  | 40.4(±37.3) | 40.5(±27.0) | 3 |
|  | 1B_II_III | 2.5 | 1.5 |  |
|  | 1B_IV_II | 20.3 | 10.4 |  |
|  | 1B_IV_III | 77.2 | 88.1 |  |
| *Theristus* Bastian 1865 |  | 35.8(±20.0) | 38.6(±28.7) | 2 |
|  | 1B_IV_II | 1.3 | 1.4 |  |
|  | 1B_IV_III | 16.9 | 26.8 |  |
|  | 1B_IV_IV | 14.3 | 5.6 |  |
|  | 1B_V_II | 2.6 | 1.4 |  |
|  | 1B_V_III | 42.9 | 40.8 |  |
|  | 1B_V_IV | 20.8 | 23.9 |  |
|  | 1B_V_V | 1.3 |  |  |
| **Sphaerolaimidae** |  |  |  |  |
| *Metasphaerolaimus* Gourbault & Boucher 1981 |  | 6.2(±7.4) | 3.7(±6.0) | 3 |
|  | 2B_I_II | 46.7 | 57.1 |  |
|  | 2B_I_II | 53.3 | 28.6 |  |
|  | 2B_I_IV |  | 14.3 |  |
| *Sphaerolaimus* Bastian 1865 |  | 11.0(±13.5) | 15.9(±12.2) | 3 |
|  | 2B_IV_I | 3.7 |  |  |
|  | 2B_IV_II | 37 | 42.9 |  |
|  | 2B_IV_III | 59.3 | 53.6 |  |
|  | 2B_IV_IV |  | 3.6 |  |
| *Subsphaerolaimus* Lorenzen 1978 |  | 0.6(±2.3) | 1.0(±2.6) | 3 |
|  | 2B_IV_III | 100 |  |  |
| **Siphonolaimidae** |  |  |  |  |
| *Astomonema* Ott, Rieger & Enderes 1982 |  | 0.6(±2.3) |  | 3 |
|  | 1A_I_III | 100 |  |  |
| *Siphonolaimus* De Man 1883 |  | 0.6(±2.3) | 2.2(±4.0) | 3 |
|  | 2B_I_III | 100 | 100 |  |
| **Linhomoeidae** |  |  |  |  |
| *Disconema* Filipjev 1922 |  |  | 0.7(±2.6) | 2 |
|  | 2B_IV_III |  | 100 |  |
| *Eleutherolaimus* Filipjev 1922 |  | 12.1(±11.3) | 26.6(±38.8) | 2 |
|  | 1B_II_III | 4 | 10 |  |
|  | 1B_II_II |  | 2.5 |  |
|  | 1B_II_III | 44 | 27.5 |  |
|  | 1B_II_IV |  | 2.5 |  |
|  | 1B_V_III | 8 | 32.5 |  |
|  | 1B_V_IV | 44 | 25 |  |
| *Eumorpholaimus* Schulz 1932 |  | 1.8(±3.7) | 1.5(±3.4) | 2 |
|  | 1B_I_II | 33.3 |  |  |
|  | 1B_I_III | 66.7 | 50 |  |
|  | 1B_IV_IV |  | 50 |  |
| *Linhomoeus* Bastian 1865 |  | 2.0(±3.6) |  | 2 |
|  | 2A_IV_III | 25 |  |  |
|  | 2A_V_III | 75 |  |  |
| *Megadesmolaimus* Wieser 1954 |  | 6.3(±9.0) | 2.5(±3.8) | 2 |
|  | 1B_I_III | 16.7 | 16.7 |  |
|  | 1B_III_II | 8.3 |  |  |
|  | 1B_IV_II | 16.7 | 16.7 |  |
|  | 1B_IV_III | 33.3 | 50 |  |
|  | 1B_IV_IV | 25 | 16.7 |  |
| *Metalinhomoeus* De Man 1907 |  | 12.4(±10.7) | 9.4(±10.8) | 2 |
|  | 1B_III_III | 7.4 |  |  |
|  | 1B_IV_III | 66.7 | 56.3 |  |
|  | 1B_IVIV | 25.9 | 43.8 |  |
| *Gen. I* indet |  | 1.2(±2.8) | 0.7(±2.6) | 2 |
|  | 1B_IV_II | 25 |  |  |
|  | 1B_IV_III | 75 | 100 |  |
| *Gen. II* indet |  | 1.4(±3.1) |  | 2 |
|  | 1B_I_II | 66.7 |  |  |
|  | 1B_I_IV | 33.3 |  |  |
| **Axonolaimidae** |  |  |  |  |
| *Ascolaimus* Ditlevsen 1919 |  |  | 0.3(±0.9) | 2 |
|  | 1B_IV_IV |  | 100 |  |
| *Axonolaimus* De Man 1889 |  | 0.6(±2.3) |  | 2 |
|  | 1B_V_II | 100 |  |  |
| **Diplopeltidae** |  |  |  |  |
| *Araeolaimus* De Man 1888 |  | 0.6(±2.3) | 1.5(±3.4) | 3 |
|  | 1A_II_III | 100 |  |  |
|  | 1A_II_IV |  | 100 |  |
| *Campylaimus* Cobb 1920 |  | 53.9(±30.1) | 55.2(±31.6) | 3 |
|  | 1B_I_I | 0.9 |  |  |
|  | 1B_I_II | 3.7 | 2.2 |  |
|  | 1B_I_III |  | 1.1 |  |
|  | 1B_II_I | 2.8 |  |  |
|  | 1B_II_II | 59.3 | 46.1 |  |
|  | 1B_II_III | 26.9 | 36 |  |
|  | 1B_II_IV |  | 3.4 |  |
|  | 1B_IV_II | 2.8 | 2.2 |  |
|  | 1B_IV_III | 2.8 | 6.7 |  |
|  | 1B_IV_IV | 0.9 | 1.1 |  |
|  | 1B_V_II |  | 1.1 |  |
| *Diplopeltula* Gerlach 1950 |  | 0.6(±2.3) | 0.3(±0.9) | 3 |
|  | 1A_IV_III |  | 100 |  |
|  | 1A_IV_IV | 100 |  |  |
| *Morlaixia* Vincx & Gourbault 1988 |  | 0.6(±2.3) | 1.0(±2.6) | 3 |
|  | 1B_II_II |  | 50 |  |
|  | 1B_II_III | 100 |  |  |
|  | 1B_II_IV |  | 50 |  |
| *Pararaeolaimus* Jensen 1991 |  | 3.4(±4.1) | 6.7(±8.3) | 3 |
|  | 1B_II_II | 100 | 90.9 |  |
|  | 1B_II_III |  | 9.1 |  |
| *Gen*. indet |  | 3.2(±6.3) | 1.0(±2.6) | 3 |
|  | 1A_I_II | 16.7 |  |  |
|  | 1A_II_III | 83.3 | 100 |  |

*****Nematodes belonging to the family Benthimermithidae are internal parasites with marine invertebrates as hosts. Generally, their mouth opening is reduced to a thin or indistinct intracuticular apical channel.

Legend

Numbers given with the genera names are mean values (± standard deviation) of subsamples from controls and treatments as numbers of individuals per 10 cm².

Numbers given with the functional groups of each genus state the presence of individuals (as percentage) within each functional group of the particular genus.

F_T_B groups: Each individual nematode is related to a functional feeding (**F**), tail (**T**) and body-shape (**B**) group. The nematodes were classified by these trait-combinations to functional **F_T_B** groups. First term refers to the feeding type, second to the tail-shape group and third to the body-shape group (cf. Table 1).

c-p values: Nematode genera are assigned to the **c**oloniser-**p**ersister (**c-p**) scale according to their *r* and *K* characteristics following Bongers (1990) and Bongers et al. (1991).
